# Lessons From the Development of the Immune Checkpoint Inhibitors in Oncology

**DOI:** 10.1177/1534735418801524

**Published:** 2018-09-19

**Authors:** Denis L. Jardim, Débora de Melo Gagliato, Razelle Kurzrock

**Affiliations:** 1 Hospital Sirio Libanes, Sao Paulo, Brazil; 2 University of California, San Diego, CA, USA

**Keywords:** immunotherapy, checkpoint inhibitors, drug development, phase I trials, FDA

## Abstract

Immunotherapies are becoming increasingly important in the treatment armamentarium of a variety of malignancies. Immune checkpoint inhibitors are the most representative drugs receiving regulatory approval over the past few years. In a recent study published in *Clinical Cancer Research*, we demonstrated that these agents are being developed faster than other prior anticancer therapies. All checkpoint inhibitors received priority review, being granted with at least one Food and Drug Administration expedited program. Hence, some of them are getting marketing approval after preliminary trials. The model continues to rely on phase I trials, designed with traditional models for dose definition, although a substantial number of patients are treated during the dose expansion cohorts. We demonstrated that efficacy and safety are reasonably predicted from the dose-finding portion of phase I trials with these agents, assuring a low treatment-related mortality for patients throughout the development process. In this article, we further discuss and summarize these findings and update some recent approval information for immune checkpoint inhibitors.

Over the past 4 years, the oncology community has faced a shift in the field of immunotherapy for cancer treatment. For many years, a first wave of attempts at boosting the host immunological system against cancer cells was concentrated in pursuing immunostimulatory agents. Vaccines, stimulatory peptides, interleukin, interferon, and adoptive lymphocytes, among others, were the subject of a myriad of clinical trials. In spite of that effort, few agents received Food and Drug Administration (FDA) approval: interferon and interleukin-2 for kidney cancer and melanoma,^1-3^ and sipuleucel-T for prostate cancer.^4^ Even in these settings, the use of immunotherapy was restricted to only a few technologically advanced cancer centers in the world due to the complexity of treatments and/or cost concerns. The advances in the understanding of the negative regulators of the host immune system against cancer led to a new era for immunotherapy in this disease. Monoclonal antibodies targeting the cytotoxic T-lymphocyte–associated antigen 4 (CTLA-4) and the programmed cell death protein pathway (PD1/PD-L1) entered clinical development, obtaining a variety of regulatory approvals.^5^ The first approval was granted for ipilimumab in March 2011 for the treatment of metastatic melanoma.^6^ Since then, another 5 checkpoint inhibitors were approved for more than 10 different types of cancers.

Despite clear differences in the mechanism of action, efficacy, and safety from traditional cytotoxic agents, targeted therapies, and first-generation immunotherapies, immune checkpoint inhibitors followed a similar development track—phase I trials to Biologic License Application approval (which marks the FDA approval). Moreover, the majority of these drugs followed an expedited development pathway, obtaining regulatory approval after initial clinical studies (such as phase Ib and phase II trials), employing surrogate endpoints as benchmarks for regulatory review.

In a recent study published in *Clinical Cancer Research*,^7^ we explored the paradigms applied for the development of the immune checkpoint inhibitors currently approved by the FDA. Our first point is that, indeed, these drugs are being developed faster than other anticancer agents approved in the past. Total time for development of approved checkpoint inhibitors reached a median of 60.77 months, which compared favorably with other anticancer agents approved between September 1999 and July 2014 (median total clinical development of 81.4 months).^8^ This timeline is more similar to targeted therapies developed under a personalized biomarker-driven strategy, for which total development took a median of 64.8 months. The acceleration in clinical development is more evident for the newer PD-1/PD-L1 inhibitors than for ipilimumab and is predicated on 2 features: all checkpoint inhibitors were included in at least one FDA expedited program and, except for nivolumab and ipilimumab, all used data from a non–phase III trial for approval. Our dataset was locked on June 1, 2017, and, as of today, no other new checkpoint inhibitor received first regulatory approval. But new indications were obtained for approved agents, including gastric and cervical cancer (pembrolizumab),^9^ hepatocellular carcinoma (nivolumab),^10^ and renal cell cancer (ipilimumab plus nivolumab).^11^ These data reinforce the amplitude of efficacy of these agents, but do not affect the timeline analysis we reported. Moreover, atezolizumab received European Medicines Agency approval on September 29, 2017. The gap between first FDA approval and European Medicines Agency approval for this drug was 16 months. Interestingly, this information complements our data since we observe a trend toward an increase in this gap more recently, which could reflex the expedited approval program led by the FDA.

One important finding from our analysis consisted of the attenuated capability of phase 1 trials to predict and define the definitive dosing and schedule used for immune agents in later trials. In fact, the later trials (that led to regulatory approval) of the immune checkpoint inhibitors adopted a dose that ranged from 50% to 400% of the recommended phase 2 dosing (RP2D) from phase I studies. We demonstrated that all the phase I trials used a traditional dose escalation design (3 + 3 escalating doses), aiming to find RP2D based on toxicities. As a result, none of the trials testing PD1/PD-L1 inhibitors reached a maximum tolerated dose and RP2D was recommended based on alternatives parameters, not included as a primary objective of the trial (such as pharmacokinetics and pharmacodynamics [PDs] parameters). As a consequence, the schedule of checkpoint inhibitors has undergone several adjustments after agent approval, as exemplified by the recent change in the label of nivolumab, including the 480-mg flat dose every 4 weeks as an alternative.^12^ Some important aspects should be considered, including the late occurrence of the immune-related toxicities, most commonly occurring after the traditional 4 weeks window of dose-limiting toxicity assessment, and the lack of a clear correlation between dosing and toxicities. Hence, the traditional phase I design should consider further adaptations to define a more precise dosing of checkpoint inhibitors, as this model is mainly based on a toxicity-efficacy correlation described for cytotoxic agents. As depicted in Figure 1, we consider a more rationale model in which parameters from PD, pharmacokinetic, and safety analysis will take part on dose definition from the initial of trials design. All these parameters could take part in a concept of a minimum effective dose, a dose that would be able to produce a PD effect with no toxicities. It is also important to consider and expand analysis of toxicities, as some of the immune-related toxicities occurs only after 8 weeks of treatment.^13^

**Figure 1.**
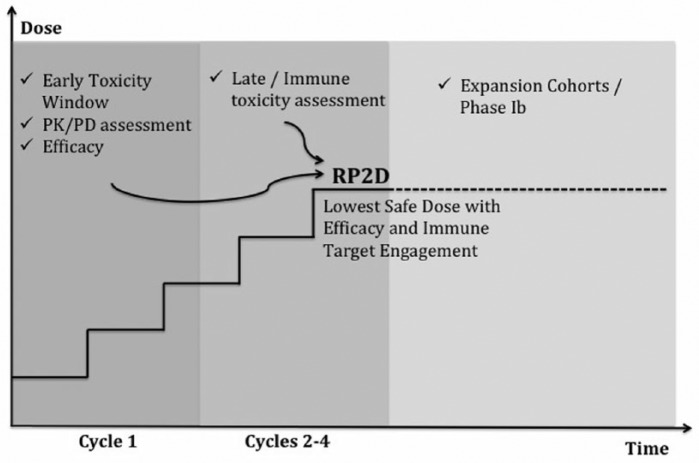
Proposed model for early development of immune checkpoint inhibitors. During the first cycle of treatment, information about early toxicity, PK/PD analysis, and efficacy is collected. Dose escalation will continue, and a second toxicity window will correlate immune toxicities with doses. Early and late assessment of information will determine the RP2D for dose expansion cohorts, which could further define safety and efficacy profile. Abbreviations: PK, pharmacokinetic; PD, pharmacodynamics; RP2D, recommended phase 2 dose.

Considering that many of the modern immunotherapies are arriving early for market access, one important concern is whether or not early trials are predictive of the toxicity profile from checkpoint inhibitors. A recent study demonstrated that cancer drugs (including 16% of immunotherapies) initially approved based on a nonrandomized trial, frequently are associated with more postmarketing safety label modifications.^14^ Emerging challenges after drug approval is also expected for immune checkpoint inhibitors, such as the recently described hyperprogression with these agents.^15,16^ Overall, we found that 50.9% of the types of immune-related toxicities detected in later trials were already evident in the phase I studies from checkpoint inhibitors. Additionally, 43% of clinically relevant types of toxicities seen in later trials were described during dose escalation portion. Although the later number is lower than the 70% prediction of clinically relevant toxicities, we previously reported for other agents (the majority cytotoxic and targeted drugs)^17^ the treatment-related mortality from phase I and later trials with checkpoint inhibitors are remarkably low (0.18% and 0.33%, respectively), reflecting the safety of the original model. Interestingly, we reported that a better description of types of immune-related toxicities is associated with more patients being included in the phase I trial. As we only considered data from the dose-escalating portion for our model, it is reasonable to believe that large dose-expansion cohorts will further enhance the toxicity prediction of immune checkpoint phase I trials.

Considering that phase I trials also have a therapeutic intent and many patients seek participation in these trials as a treatment option,^18,19^ we also sought to explore the efficacy paradigm for checkpoint inhibitors. Overall, response rate (RR) obtained only from the dose-escalating portion was 16%, which is higher than the historical RR of 5% for genomically targeted agents or chemotherapy performed without a biomarker.^20^ This RR is very close to the 20% benchmark we described for oncology drug successful development during phase II trials.^21^ Interestingly, we reported that frequently the phase I trials mirror the RR of the later trials for each indication, and we described the absolute difference in RR from these comparisons ranging from 9% to 18%. Additionally, in 3 of 8 (37.5%) comparisons, the RR from phase I was higher compared with the later trial.

Overall, the current drug development of immune checkpoint inhibitors highlights an important effort in getting drug approvals faster, frequently using expedited programs especially by the FDA. Although using a trial design and a development strategy classically created for cytotoxic agents, the current model assures safety and efficacy for patients with cancer throughout the process, justifying the rapid approvals. Nonetheless, the model could use some innovative adjustments, especially in the search for new strategies for drug combinations and a more precise dose definition after initial phase I trials.
